# Supplementary material for: Sex-converted testis somatic cells acquire female-specific behaviors and alter XY germline identity
Source: Development. 2025 Aug 7;152(15):dev204785. doi: 10.1242/dev.204785 (PMC12377807; doi:10.1242/dev.204785)
Supplement: Supplementary information [file develop-152-204785-s1.pdf]

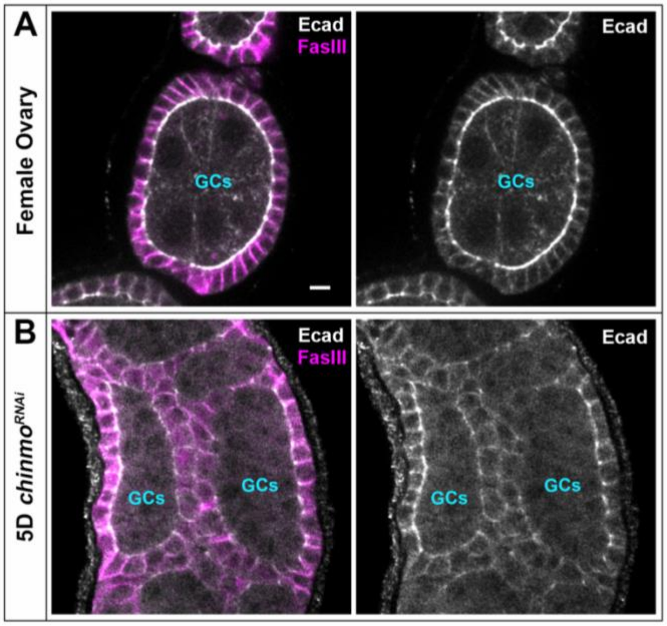

Fig. S1. Loss of Chinmo induces formation of a polarized somatic epithelium similar to female follicle cells.

(A-B) Immunofluorescent staining of Ecad (grey) and Fas3 (magenta) in a (A) control ovary and (B) 5D *chinmo*^{RNAi} testis, both showing apical localization of Ecad towards germ cells. Germ cells (GCs) are indicated. Scale bar: 5 µm. Each image is 1 z-slice.

Development • Supplementary information

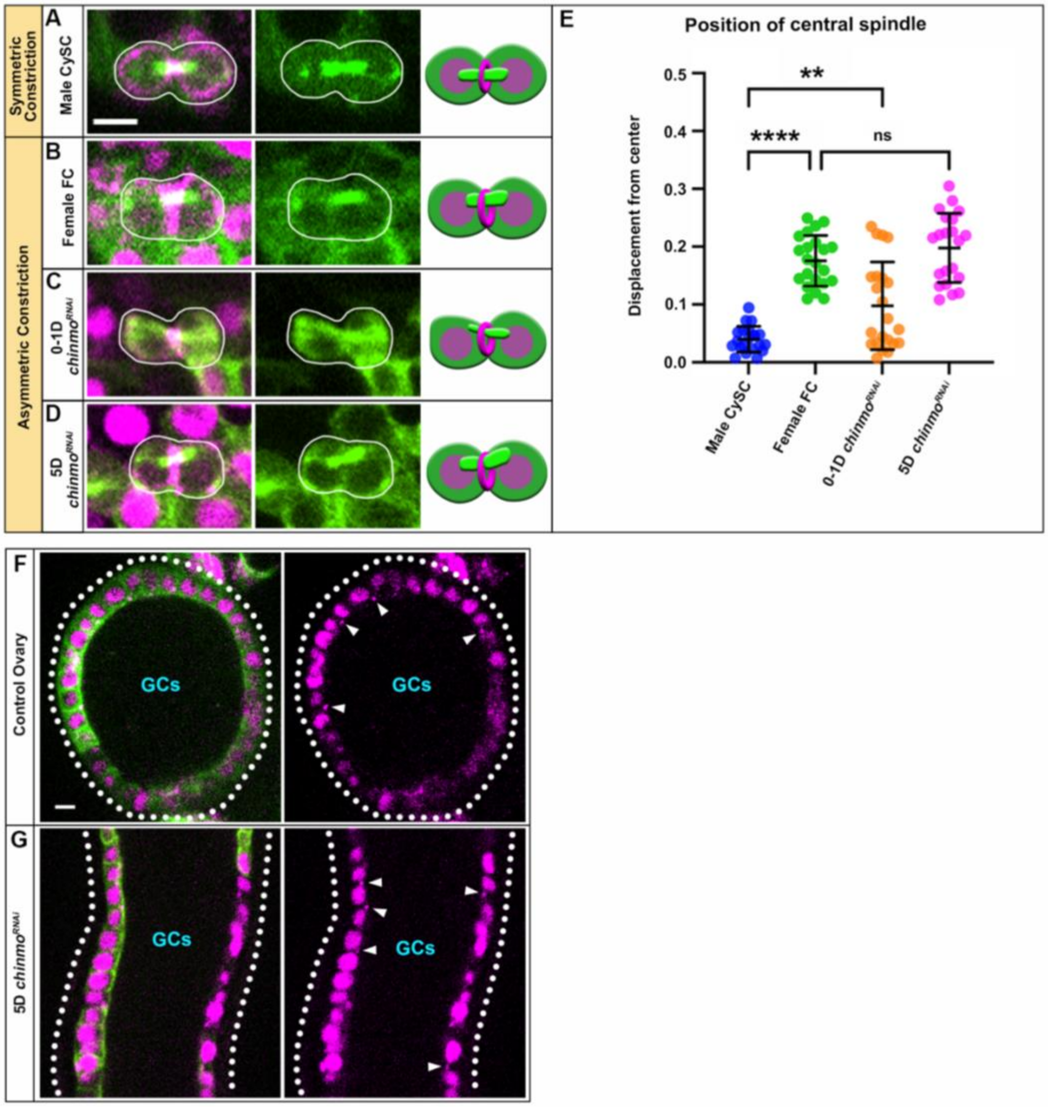

Fig. S2. Male somatic cells initiate conserved, epithelia-specific cell division behaviors upon loss of Chinmo.

(A-D) Stills from live imaging of somatic tubulin (green) and anillin (magenta) during constriction of the AMC ring in a (A) male CySC, (B) female FC, (C) 0-1D *chinmo*^{RNAi} somatic cell, and (D) 5D *chinmo*^{RNAi} somatic cell. (E) Quantification of displacement of the center of the furrow ($n \geq 21$ cells in 7 samples). **p<0.0047, ****p<0.0001 (One-way ANOVA). Ns, not significant. Error bars: standard deviation of the mean. (F-G) Stills from live imaging of somatic tubulin (green) and anillin (magenta) showing apical localization of midbodies (arrowheads) in a (F) control ovary and (G) 5D *chinmo*^{RNAi} testis. Dotted outline marks basal periphery of the tissue. Germ cells (GCs) are indicated. All experiments $n \geq 2$ trials. Scale bar: 5µm (for A-D, F-G). Each image is 1-4 z-slices.

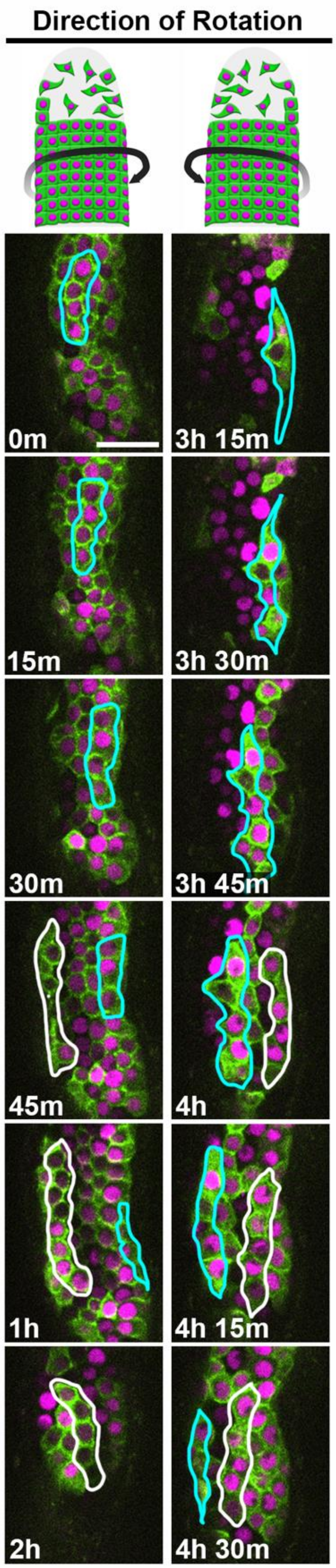

Development • Supplementary information

Fig. S3. The somatic epithelium in *chinmo*^{RNAi} testes can reverse migration direction.

Top: Diagrams indicating direction of epithelial rotation within the same testis over time. Bottom: Stills from live imaging of somatic tubulin (green) and anillin (magenta) in a 5D 5D *chinmo*^{RNAi} testis. Left panels: The somatic epithelium initially migrates in a “left to right” or clockwise direction. Right panels: Over time, the somatic epithelium changes the direction of migration and now moves in a “right to left” or counterclockwise direction. Scale bar: 10µm. Each image is 1 z-slice. Part of this figure was created in BioRender by Roach, T. 2025. https://BioRender.com/pqfur6k. This figure was sublicensed under CC BY 4.0 terms.

Development • Supplementary information

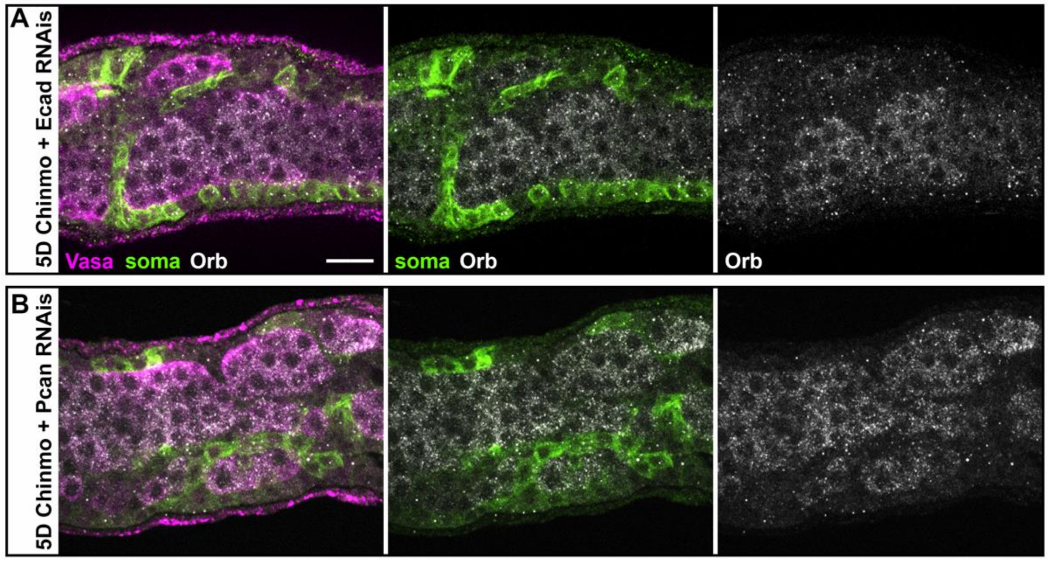

Fig. S4. Retention of Orb induction upon combined loss of Chinmo and Ecad or Pcan.

(A) Orb induction in germ cells surrounded by a disrupted somatic epithelium in 5D *chinmo*^{RNAi} + *Ecad*^{RNAi}. (B) Orb induction in germ cells surrounded by a somatic epithelium in 5D *chinmo*^{RNAi} + *Pcan*^{RNAi}. All experiments n≥2 trials. Each image is 1-3 z-stacks. Scale bar: 20µm.

Table S1. Reagents and resources

REAGENT or RESOURCE	SOURCE	IDENTIFIER
Antibodies		
Rat anti-DE-cadherin	DSHB	RRID: AB 528120
Guinea pig anti-Traffic Jam	Dorothea Godt	N/A
Rabbit anti-Vasa	Boster Biological Technology Co.	RRID: DZ 41154
Chicken anti-GFP	Aves Labs	RRID: AB 10000240
Mouse anti-fasciclin III	DSHB	RRID: AB 528238
Mouse anti-Orb	DSHB	RRID: AB 528419
Mouse anti-Bicaudal-D	DSHB	RRID: AB 528102
Experimental models: Organisms/strains		
D. melanogaster: Tj-Gal4	Kyoto Stock Center	104055
D. melanogaster: nanos-lifeact::tdTomato	Lehmann Lab	N/A
D. melanogaster: UAS-chinmo-RNAi	Bloomington *Drosophila* Stock Center	BDSC: 33638 FBti0140111
D. melanogaster: UAS-Scra::mRFP	Bloomington *Drosophila* Stock Center	BDSC: 52220 FBti0156510
D. melanogaster: UAS-tubulin::GFP	Bloomington *Drosophila* Stock Center	BDSC: 7374 FBti0038622
D. melanogaster: UAS-Ecad-RNAi	Bloomington *Drosophila* Stock Center	BDSC: 38207 FBgn0003391
D. melanogaster: UAS-Perelcan-RNAi	Vienna *Drosophila* Resource Center	RRID:SCR 24549
D. melanogaster: Zip::GFP	*Drosophila* Genomics Resource Center	RRID:CVCL_Z854 FBtc0000194
Software and algorithms		
ImageJ/Fiji	https://fiji.sc	RRID:CVCL_0299
Graphpad Prism	https://www.graphpad.com/scientific-software/prism/	RRID:SCR_002798
Adobe Photoshop	https://www.adobe.com/uk/products/photoshop.html	RRID:SCR_014199
Imaris 10.2 (Bitplane, Oxford, UK)	https://imaris.oxinst.com/products/imaris-for-core-facilities	Imaris for Core Facilities; Oxford Instruments
Other		
Vectashield	Vector laboratories	Cat# H-1200
Imaging dishes with coverslip bottom	MatTek	Cat# P35G-1.5-14-C
Olympus iX83 inverted spinning disk confocal	Olympus/Evident	N/A
Hamamatsu EM-CCD camera	Olympus/Evident	N/A
60X 1.4NA silicon oil immersion objective	Olympus/Evident	N/A
Leica Stellaris 5 DMi8 inverted stand with tandem scanner and 4 power HyD spectral detectors	Leica	N/A
HC PL APO 63X 1.4NAA CS2 oil objective	Leica	N/A
Zeiss 700 confocal microscope	Zeiss	N/A

Development • Supplementary information

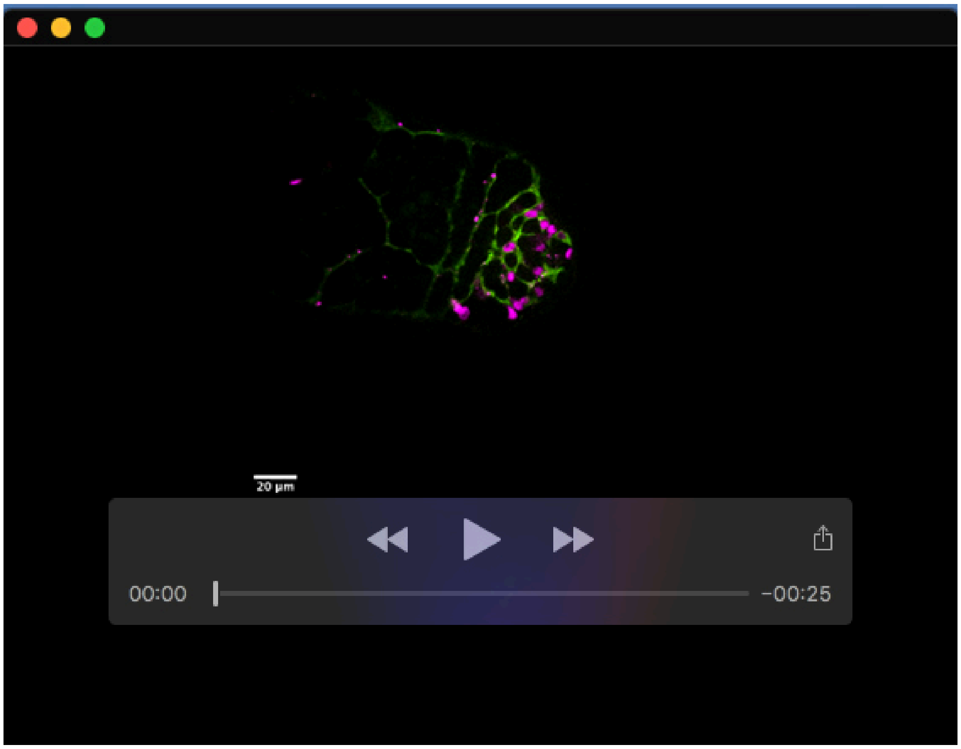

Movie 1. Timelapse of control testis somatic cells. Live imaging of a single z-plane visualizing tubulin::GFP (green) and anillin::RFP (magenta). Somatic cells move independently and slowly with no directionality.

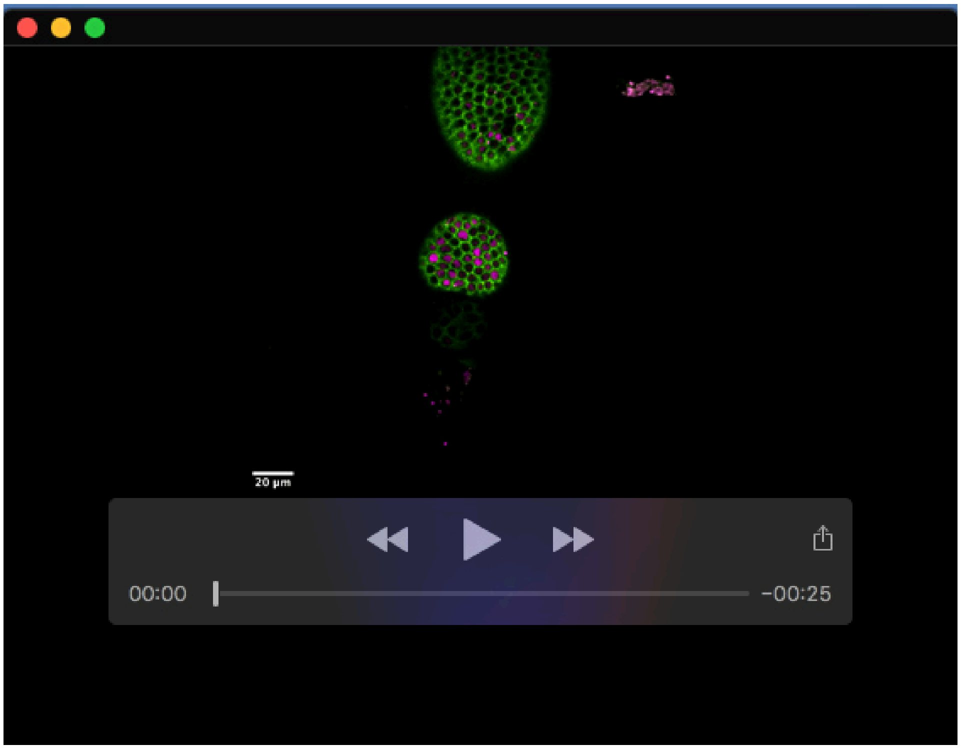

Movie 2. Timelapse of control ovarian somatic cells. Live imaging of a single z-plane visualizing tubulin::GFP (green) and anillin::RFP (magenta). Somatic cells exhibit rotational migration as an epithelium around the underlying germ cells.

Development • Supplementary information

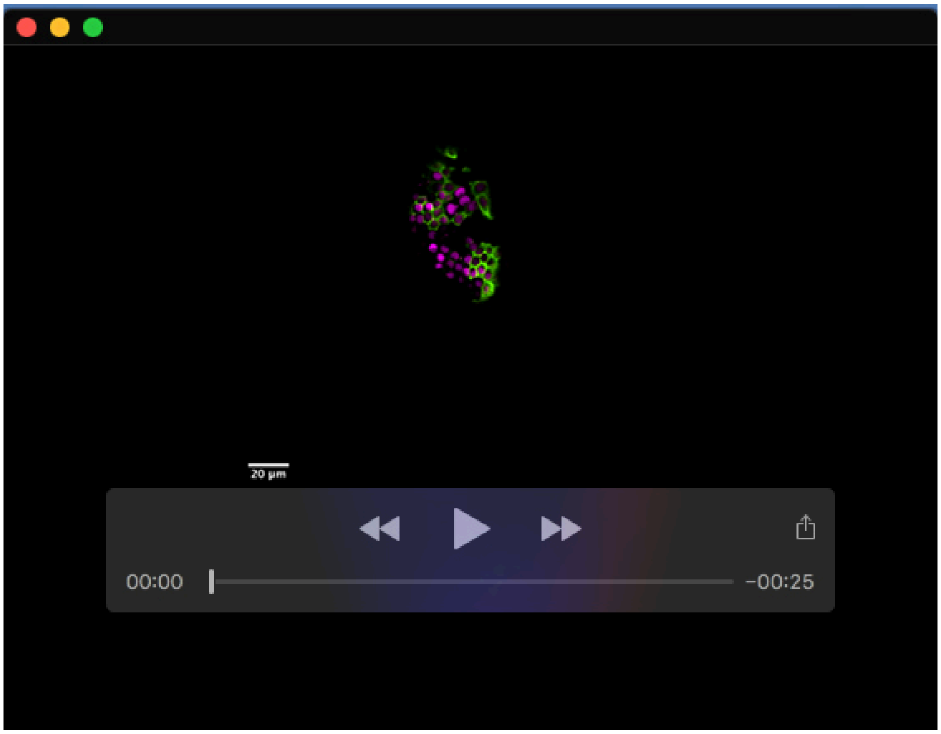

Movie 3. Timelapse of Chinmo-depleted testis somatic cells. Live imaging of a single z-plane visualizing tubulin::GFP (green) and anillin::RFP (magenta). *chinmo*-deficient somatic cells induce female-specific rotational migration as an epithelium around underlying germ cells.

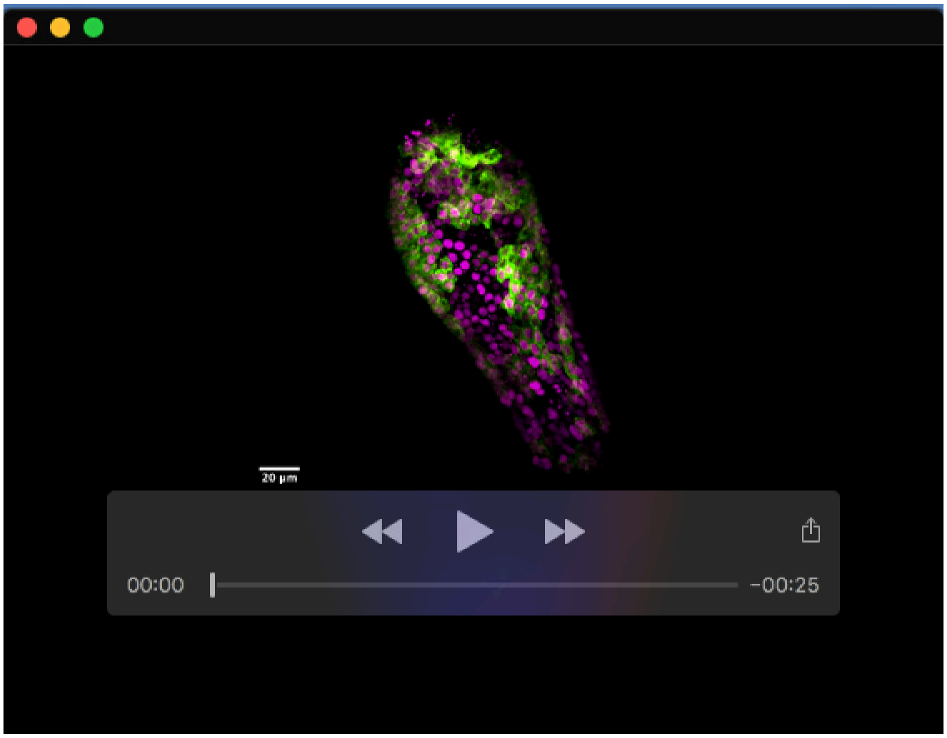

Movie 4. Tissue-level view of soma rotational migration in Chinmo-depleted testis. 3D projection of Supplemental Video 3.

Development • Supplementary information

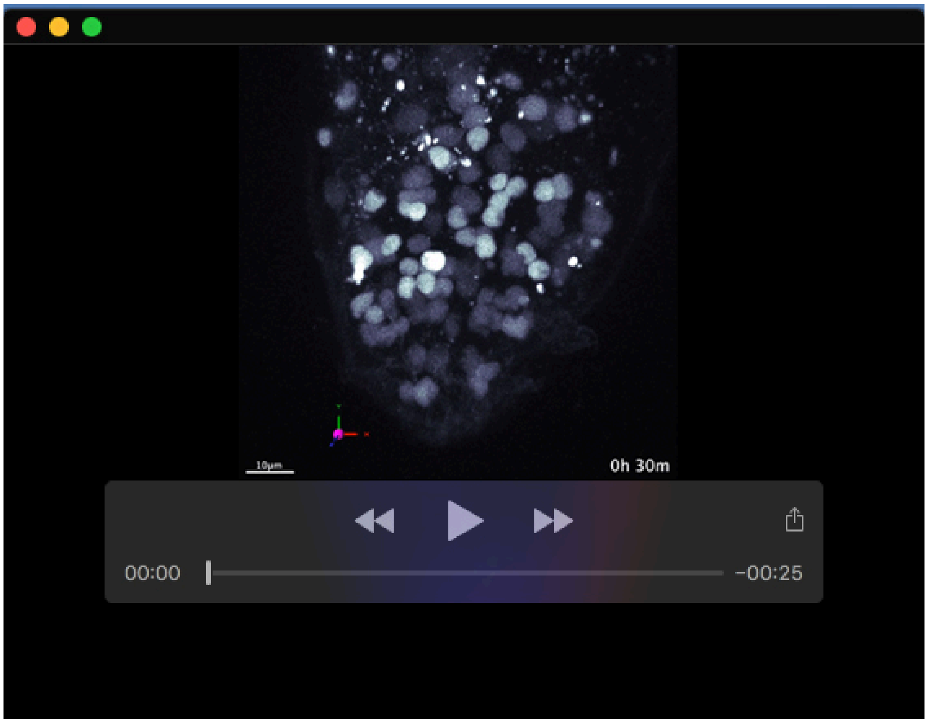

Movie 5. Somatic nuclear tracking in control testis. Control testis somatic nuclei (marked by colored dots) move independently with no directionality (marked by colored tracks) with minor displacement (red arrows).

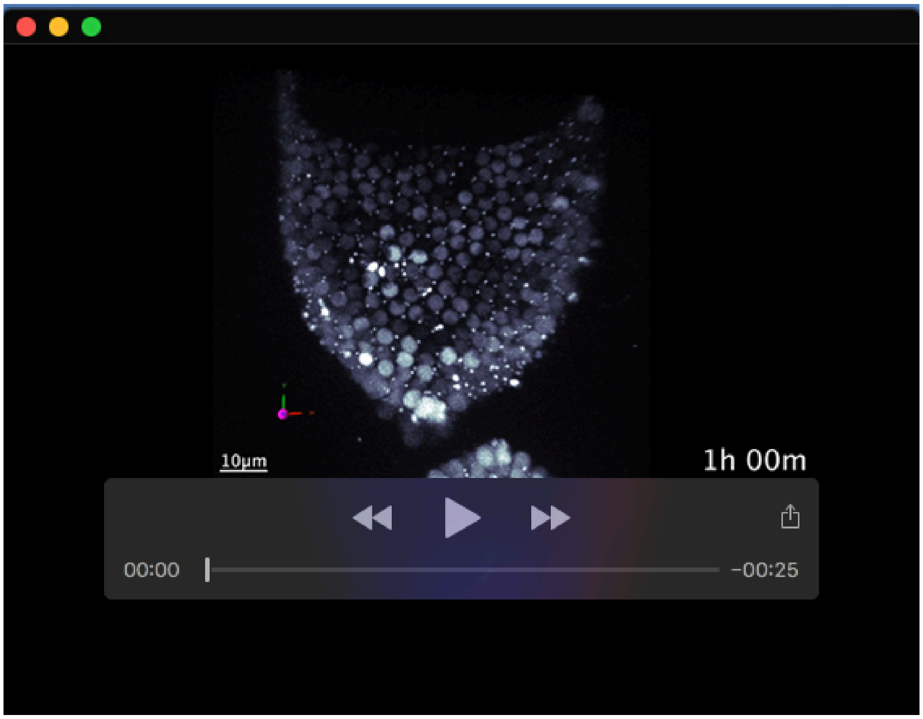

Movie 6. Somatic nuclear tracking in control ovary. Control ovarian somatic nuclei (marked by colored dots) move coordinately with consistent directionality (marked by colored tracks) and significant displacement (red arrows).

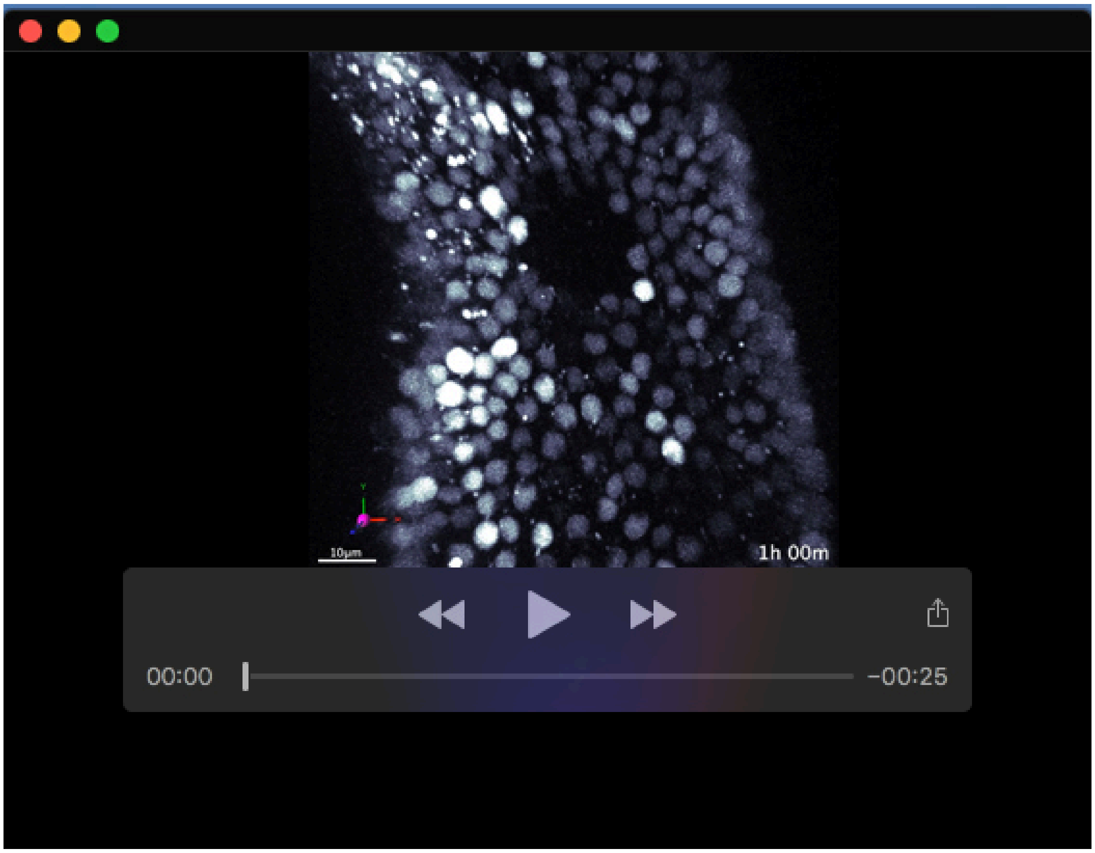

Movie 7. Somatic nuclear tracking in Chinmo-depleted testis. Somatic nuclei in *chinmo*-deficient cells (marked by colored dots) move coordinately with consistent directionality (marked by colored tracks) and significant displacement (red arrows).
